# Sex Differences in Hypercortisolism and Glucose-Metabolism Disturbances in Patients with Mild Autonomous Cortisol Secretion: Findings From a Single Center in China

**DOI:** 10.3389/fendo.2022.857947

**Published:** 2022-06-09

**Authors:** Ru Ouyang, Yaqi Yin, Jie Wang, Wanlu Su, Li Zang, Kang Chen, Jin Du, Zhaohui Lyu, Jingtao Dou, Yiming Mu, Weijun Gu

**Affiliations:** ^1^ Department of Endocrinology, the First Medical Center of Chinese People’s Liberation Army General Hospital, Beijing, China; ^2^ Department of Endocrinology, Sanya Central Hospital, Sanya, China; ^3^ Department of Endocrinology, Hainan Affiliated Hospital of Hainan Medical University, Hainan General Hospital, Haikou, China; ^4^ Department of Endocrinology, Beijing Chao-yang Hospital, Beijing, China; ^5^ School of Medicine, Nankai University, Tianjin, China

**Keywords:** hypercortisolism, autonomous cortisol secretion, subclinical Cushing’s disease, sex differences, menopausal status

## Abstract

**Background and objective:**

Mild autonomous cortisol secretion (MACS) presents with a marked female preponderance, but whether the sex difference in its distribution has any relevance to the presentation and outcome of the disease is unknown. The aim of this study was therefore to compare biochemical indices of hypercortisolism and impaired glucose metabolism between male and female patients with MACS.

**Method:**

We enrolled a total of 98 patients with autonomous/possible autonomous cortisol secretion in our study, and indices of hypercortisolism and glucose metabolism were collected and compared between the male and female patients. Logistic regression models were used to evaluate the association between sex and cortisol-secretory ability, as well as between the latter and glucose metabolism. In addition, we conducted further stratified analyses according to the degree of autonomous cortisol secretion and menopausal status.

**Results:**

Cortisol levels at 00:00 and 08:00 h after a 1-mg dexamethasone suppression test (DST) and low-dose DST were significantly higher in female than in male MACS patients, and the inhibition rate of 1-mg DST was lower in the women than in the men. This significant difference still remained after adjusting for age, BMI, and the course of the disease. Logistic regression analysis revealed a significant association between autonomous cortisol secretion and fasting C-peptide, as well as with the C-peptide-to-glucose ratio in females relative to male patients. In addition, stratified analyses indicated that this association was observed only among women with autonomous cortisol secretion and who were premenopausal.

**Conclusion:**

The level of autonomic cortisol secretion in female patients with MACS was higher than in male patients, and the association between autonomous cortisol secretory ability and glucose homeostasis was only noted in patients with autonomous cortisol secretion and in premenopausal women. This phenomenon will, however, require closer follow-up.

## Introduction

Adrenal incidentalomas (AIs) are defined as unsuspected adrenal masses discovered during imaging procedures conducted for non-adrenal-related reasons, and that are increasingly encountered in clinical practice because of continued advances in imaging technology and easier access to imaging modalities. AIs are considered common, with reported prevalence rates varying between 2.3% and 5.5% in different analyses (1–3). The discovery of AIs engenders evaluations of autonomous hormone hypersecretion, and in this phenomenon, 5–30% (4) of cases demonstrate subtle cortisol overproduction without classical signs or symptoms of overt cortisol excess; this is commonly referred to as subclinical Cushing’s syndrome (SCS), hidden hypercortisolism (HydHyCo), or mild autonomous cortisol secretion (MACS) as per ESE guidelines is recommended. In a previous study from our center, we noted this proportion to be at 7.1% (5). Apart from adrenal origin, what cannot be ignored is that, MACS also involves a 5% prevalence of ACTH-secreting pituitary adenomas (6). Although MACS is not associated with symptoms specific to Cushing’s syndrome such as purple striae or proximal muscle weakness, evidence indicates that long-term, subtle cortisol excess can affect glucose metabolism and lead to an increased prevalence of insulin resistance (7), and that surgical treatment may bring about a favorable outcome (8–11).

Many endocrine disorders show sex differences in disease prevalence, clinical manifestations, laboratory findings, and prognosis (12). Similar to Cushing’s disease (13), data suggest that MACS also exhibits a marked female preponderance (14). However, apart from epidemiological considerations, whether this sexual dimorphism carries over into clinical manifestations and biochemical indices of MACS has rarely been evaluated, and a possibly skewed sex influence on the association between cortisol autonomous secretion and glucose metabolism remains uninvestigated. Therefore, it is important to investigate such potentially unrecognized sex differences, and in this clinical study we analyzed the data from a tertiary hospital in China and performed separate evaluations of male and female patients instead of referring to the population as a whole.

## Subjects and Methods

This retrospective study was conducted at the Endocrinology Department of the Chinese PLA General Hospital, Beijing, China. The study was approved by the ethics committee of Chinese PLA General Hospital (NO. S2021-555-01). A waiver of the requirement to obtain informed consent from the study subjects was approved considering the minimal risk of the study.

### Study Population

The subjects were patients with MACS who were hospitalized because of AIs between January 2010 and December 2019. In accordance with the 2016 European Society of Endocrinology (ESE) guideline on AIs (15), 122 subjects without external manifestations of overt Cushing’s syndrome (such as purple striae, plethoric face, buffalo hump, and ecchymoses) but with serum cortisol levels of >50 nmol/L (>1.8 μg/dL) after a 1-mg dexamethasone test were included (and defined as having MACS). All subjects met the following conditions: 1) a disordered circadian rhythm of cortisol secretion and 2) post-low-dose dexamethasone test (LDDST, 0.5 mg/6 h for 48 h) cortisol levels ≥50 nmol/L. Subjects meeting the following criteria were excluded: 1) an absence of information on basic indices such as 24-h urine-free cortisol (24 h UFC), HbA1c, and fasting C-peptide; 2) the presence of congenital adrenal hyperplasia (CAH), pheochromocytoma, primary aldosteronism (PA), or malignancy; and 3) having other known conditions that may affect the level of those indices related to cortisol and glucose metabolism, such as severe chronic hepatic disease, chronic renal failure (eGFR <50 mL/min), alcoholism, depression, medication history of exogenous glucocorticoids, or other drugs that affect the hypothalamic-pituitary axis taken within one-half year before admission. After these exclusions, the remaining study population with autonomous cortisol secretion was comprised of 98 participants (33 men and 65 women). Subjects with serum cortisol levels between 51 and 138 nmol/L post-1-mg dexamethasone test were defined as the low-cortisol group (LCG), and the remainder with cortisol levels post-dexamethasone of >138 nmol/L were designated as the high-cortisol group (HCG).

### Clinical Examinations and Definitions

Composite diabetes included patients with a documented diagnosis of type 2 diabetes (T2D) and therapy with at least one glucose-lowering medication prior to admission to the hospital. Body mass index (BMI) was calculated as weight/height squared (in kg/m^2^). All patients were admitted to a standard hospital ward after a history and physical examination and received biochemical and endocrinological testing. To avoid stress, a sampling catheter was placed, and an endocrinological evaluation was performed on the second day of hospitalization. The ward turns off the lights at 10 p.m. and turns them on at 6 a.m. All subjects underwent biochemical evaluations that consisted of a 24 h UFC and a serum cortisol (F) and plasma ACTH at 00:00, 08:00, and 16:00 h - followed by a 1-mg overnight dexamethasone suppression test (1-mg DST) and measurement of serum cortisol and plasma ACTH levels at 08:00 the next morning. The inhibition rate of 1-mg-DST was calculated as the (serum cortisol at 08:00 minus the serum cortisol 08:00 post-1-mg DST)/serum cortisol at 08:00. Patients with cortisol levels ≥50 nmol/L post-1-mg DST underwent an additional LDDST. We also evaluated the glucose-metabolism indices of fasting plasma glucose (FPG), fasting C-peptide (FCP), and HbA1c. The fasting C-peptide-to-glucose ratio (fCGR) was calculated as the ratio of fasting serum C-peptide to fasting serum glucose. The imaging technique applied to AI was not standardized, and the data from CT, MRI, and ultrasonography were used for mass localization and estimation of size.

### Laboratory Measurements

Cortisol and ACTH levels were measured by chemiluminescence immunoassays and were analyzed using an ADVIA Centaur Analyzer (Siemens Healthcare Diagnostics, Tarrytown, NY, USA) and an Immulite 2000 Analyzer (Siemens Health-care Diagnostics Inc., LA, USA), respectively. Plasma glucose levels were evaluated using an auto-analyzer (Cobas 8000 Modular Analyzer series; Roche Diagnostics, Basel, Switzerland), and FCP and HbA1c levels were determined with a high-performance liquid chromatography method using the VARIANT II Hemoglobin Testing System (Tosoh Corporation, Tokyo, Japan). All tests were performed in the Biochemistry Department and the Endocrine Laboratory of the Chinese PLA General Hospital.

### Statistical Analysis

Data are presented as mean ± standard deviation (SD) or median (interquartile range) for continuous variables, and as frequency and percentage for categorical variables. For the analysis of baseline characteristics, the differences between men and women were examined using a t-test for data following a normal distribution or the Kruskal–Wallis rank-sum test for non-normally distributed continuous variables and the Chi-squared test for categorical data. For further analysis, we exploited logistic regression models to evaluate the association between cortisol inhibition rate and the status of glycemic metabolism between men and women. Unadjusted and multivariate adjusted models were applied, and β and 95% confidence intervals (CI) were calculated. We also conducted stratified analyses according to patient state with respect to PACS or ACS, and menopausal status.

All statistical analyses were implemented using the R statistical software package (http://www.R-project.org; The R Foundation) and EmpowerStats (http://www.empowerstats. com; X&Y Solutions, Inc., Boston, MA, USA). A two-tailed significance level of 0.05 was used to evaluate statistical significance.

## Results

**Figure 1:**
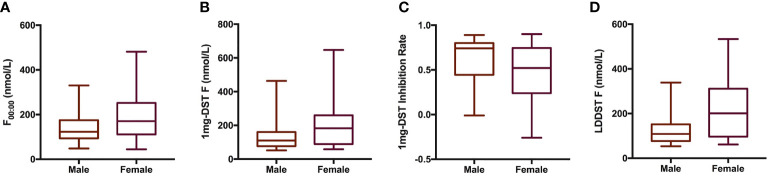
Cortisol-related indices of male and female patients with MACS. Box plots presented cortisol related indices including F_00:00_ **(A)**, 1mg-DST F **(B)**, 1mg-DST Inhibition Rate **(C)** and LDDST **(D)** of male (n = 33) and female (n = 65) patients with MACS. The blank bar within the box represents the median. The box refers to the upper (^75^th percentile) and lower (^25^th percentile) hinges. Maximum and minimum values are represented by whiskers. F_00:00_, cortisol level at 00:00; 1mg DST, 1-mg overnight dexamethasone test; LDDST, low-dose dexamethasone test.

We included a total of 98 patients with MACS in this study, 33 men and 65 women, with a male-to-female ratio of 1:1.97 (**Table 1**). There were no significant differences in age, course of disease, tumor location or diameter between men and women. The BMI of male patients showed a tendency to be slightly higher than that of females, with a *P* value of 0.07. In terms of cortisol rhythm, no significant differences in F_08:00_ or F_16:00_ levels were observed, although trough cortisol level, F_00:00_, of female MACS patients was significantly higher than that of males (**Figure 1A**). The cortisol levels of female MACS patients after a 1-mg DST and LDDST were significantly higher than those of male MACS patients, and the cortisol-inhibition rate of 1-mg DST in women was also lower than that for men (**Figures 1B–D**). There were no significant differences in ACTH levels at any timepoint between the sexes. In order to further substantiate the difference in autonomous cortisol secretion between male and female MACS patients, we exploited logistic regression analysis. After adjusting for age, BMI, and disease course, our results showed that the differences in the above indices were still significant between men and women (**Table 2**).

**Table 1:** Clinical characteristics and biochemical indices of MACS patients in relation to sex differences.

	Total	Male	Female	*P*-value
n	98	33	65	
Age (years, x ± s)	51.1 ± 10.3	52.2 ± 11.4	50.5 ± 9.8	0.455
BMI (kg/m^2^, x ± s)	26.1 ± 3.5	27.0 ± 3.5	25.7 ± 3.4	0.070
Course of disease (months, M[Q1, Q3])	1.0 (0.5-3.0)	1.0 (0.8-3.0)	1.0 (0.5-3.0)	0.285
Tumor Location (n (%))				0.260
Left adrenal	34 (34.7%)	10 (30.3%)	24 (36.9%)	
Right adrenal	41 (41.8%)	12 (36.4%)	29 (44.6%)	
Bilateral adrenal	23 (23.5%)	11 (33.3%)	12 (18.5%)	
Tumor diameter (mm, M[Q1, Q3])	19.0 (14.2-24.8)	18.8 (14.0-28.2)	19.0 (14.7-23.0)	0.454
ACTH_00:00_ (pmol/L, M[Q1,Q3])	1.1 (1.1-1.6)	1.1 (1.1-1.2)	1.1 (1.1-1.7)	0.140
ACTH_08:00_ (pmol/L, M[Q1,Q3])	2.4 (1.5-3.4)	2.8 (1.9-3.7)	2.2 (1.3-3.4)	0.897
ACTH_16:00_ (pmol/L, M[Q1,Q3])	1.5 (1.1-2.3)	2.0 (1.2-2.5)	1.3 (1.1-2.1)	0.723
F_00:00_ (nmol/L, M[Q1,Q3])	142.0 (101.7-228.1)	122.5 (97.3-158.9)	170.7 (114.7-250.0)	**0.006**
F_08:00_ (nmol/L, M[Q1,Q3])	383.2 (309.9-457.6)	384.0 (310.0-461.8)	382.4 (309.9-445.2)	0.910
F_16:00_ (nmol/L, M[Q1,Q3])	252.9 (195.8-322.5)	243.2 (197.8-345.0)	256.9 (174.2-304.4)	0.403
24 h UFC (μg/24 h, M[Q1, Q3])	411.8 (307.7-586.0)	496.9 (360.5-667.3)	404.9 (298.7-516.3)	0.153
1mg-DST ACTH (pmol/L, M[Q1,Q3])	1.1 (1.1-1.6)	1.1 (1.1-1.5)	1.1 (1.1-1.7)	0.236
1mg-DST F (nmol/L, M[Q1,Q3])	144.8 (82.8-230.3)	109.3 (77.9-155.0)	182.5 (89.4-256.7)	**0.013**
1mg-DST Inhibition Rate (M [Q1, Q3])	0.6 (0.3-0.8)	0.7 (0.5-0.8)	0.5 (0.2-0.7)	**0.020**
LDDST ACTH (pmol/L, M[Q1,Q3])	1.1 (1.1-1.4)	1.1 (1.1-1.3)	1.1 (1.1-1.5)	0.270
LDDST F (nmol/L, M[Q1,Q3])	156.3 (82.1-262.2)	108.4 (77.7-149.2)	201.2 (98.1-295.9)	**0.001**
HbA1c (%, x ± s)	6.0 ± 1.3	6.1 ± 1.0	6.0 ± 1.5	0.610
FPG (mmol/L, x ± s)	5.3 ± 1.4	5.5 ± 1.4	5.2 ± 1.5	0.320
FCP (mU/L, M[Q1,Q3])	2.5 (2.0-3.3)	3.2 (2.1-3.7)	2.4 (1.9-3.0)	0.114
fGCR (mU/mmol, M [Q1, Q3])	0.5 (0.4-0.6)	0.6 (0.5-0.7)	0.5 (0.4-0.6)	0.108
Diabetes (n (%))	24 (24.5%)	11 (33.3%)	13 (20.0%)	0.147

Continuous data are shown as mean ± standard deviation or median (interquartile range), and categorical data are shown as frequency (%). P-values < 0.05 are shown in bold.

BMI, body mass index; F_00:00_, cortisol level at 00:00; F_08:00_, cortisol level at 08:00; F_16:00_, cortisol level at 16:00; UFC, urine-free cortisol level; 1-mg DST, 1-mg overnight dexamethasone test; LDDST, low-dose dexamethasone test; FPG, fasting plasma glucose; FCP, fasting C-peptide; fCGR, fasting C-peptide-to-glucose ratio.

**Table 2:** Logistic regression used to analyze the difference in autonomous cortisol secretion between male and female MACS patients.

	F_00:00_			1-mg-DST F			1-mg-DST Inhibition Rate		
	β	95%CI	*P*-value	β	95%CI	*P*-value	β	95%CI	*P*-value
Unadjusted	56.9	17.1, 96.7	**0.006**	61.6	13.9, 109.3	**0.013**	-0.2	-0.3, -0.0	**0.020**
Model I	50.2	10.8, 89.6	**0.014**	52.9	5.9, 99.8	**0.030**	-0.1	-0.2, -0.0	**0.028**
Model II	48.7	9.4, 88.0	**0.017**	52.1	4.9, 99.3	**0.033**	-0.1	-0.2, -0.0	**0.028**

Model I adjusted for age and BMI. Model II adjusted for age, BMI, and course of disease.

F_00:00_, cortisol level at 00:00; 1-mg DST F, cortisol post-1-mg overnight dexamethasone test.

P-values < 0.05 are shown in bold.

In view of the effects of cortisol on glucose metabolism, we then compared whether there were sex differences in HbA1c, FPG, FCP, and fCGR and discussed the association between autonomous cortisol secretion level and glucose metabolism. **Table 1** depicts no significant sex difference in glycometabolic indices neither among MACS patients nor in the prevalence of diabetes. In **Table 3**, we noted that autonomous cortisol secretion (cortisol-inhibition rate) did not show a significant correlation with glucose-metabolism indices of the total study population when no factors were adjusted; however, the correlations between autonomous cortisol secretion levels and FCP and fCGR were apparent after adjusting for age, BMI, course of disease, and history of diabetes-especially after stratification for sex. This association was only reflected in female MACS patients; i.e., with the decrease in autonomous cortisol secretory ability, the levels of FCP and fCGR in female MACS patients diminished significantly. We further subdivided the population into low-cortisol group (LCG) and high-cortisol group (HCG) according to whether the cortisol level after a 1-mg DST was greater than 138 nmol/L. Stratified analysis showed that after adjustment for age, BMI, course of disease, and diabetes history, the associations between cortisol-inhibition rate and FCP and fCGR only appeared in the high-cortisol group of female patients (**Table 4**).

**Table 3:** Logistic regression used to analyze the association between 1-mg DST inhibition rate and glucose metabolism in men and women.

	Total			Male			Female		
FCP	β	95% CI	*P*-value	β	95% CI	*P*-value	β	95% CI	*P*-value
Unadjusted	-0.8	-2.2, 0.7	0.319	-0.2	-4.1, 3.6	0.901	-1.0	-2.2, 0.3	0.153
Model I	-1.6	-3.2, -0.0	0.050	-1.0	-5.8, 3.8	0.679	-1.8	-3.0, -0.6	**0.004**
Model II	-1.6	-3.2, -0.0	**0.049**	-1.1	-5.9, 3.7	0.671	-1.9	-3.0, -0.7	**0.003**
Model III	-1.6	-3.2, -0.0	0.051	-0.9	-5.7, 3.9	0.718	-1.9	-3.1, -0.7	**0.004**
**fGCR**	β	95% CI	*P*-value	β	95% CI	*P*-value	β	95% CI	*P*-value
Unadjusted	-0.2	-0.5, 0.1	0.277	0.0	-0.9, 0.8	0.914	-0.2	-0.4, 0.0	0.064
Model I	-0.3	-0.6, 0.0	0.090	-0.3	-1.3, 0.8	0.638	-0.3	-0.5, -0.1	**0.010**
Model II	-0.3	-0.6, 0.0	0.090	-0.3	-1.3, 0.8	0.632	-0.3	-0.5, -0.1	**0.009**
Model III	-0.3	-0.6, 0.0	0.084	-0.2	-1.2, 0.8	0.685	-0.3	-0.5, -0.1	**0.006**

Model I adjusted for age and BMI. Model II adjusted for age, BMI, and course of disease. Model III adjusted for age, BMI, course of disease, and history of diabetes.

FCP, fasting C-peptide; fCGR, fasting C-peptide-to-glucose ratio.

P-values < 0.05 are shown in bold.

**Table 4:** Logistic regression used to evaluate the association between 1-mg DST inhibition rate and glucose metabolism in men and women as stratified by level of cortisol secretion.

	Male						Female					
	LCG			HCG			LCG			HCG		
FCP	β	95% CI	*P*-value	β	95% CI	*P*-value	β	95% CI	*P*-value	β	95% CI	*P*-value
Unadjusted	1.5	-12.2, 15.2	0.831	-2.8	-6.8, 1.1	0.212	1.2	-4.1, 6.4	0.666	-2.7	-4.7, -0.7	**0.014**
Model I	1.4	-13.9, 16.7	0.859	-2.3	-6.7, 2.1	0.365	-1.8	-7.1, 3.5	0.505	-3.0	-4.9, -1.0	**0.007**
Model II	1.9	-13.7, 17.5	0.818	-2.6	-8.1, 2.9	0.427	-3.0	-8.4, 2.5	0.303	-2.9	-4.9, -1.0	**0.008**
Model III	3.7	-11.5, 18.9	0.644	-2.3	-9.8, 5.1	0.598	-3.0	-8.6, 2.6	0.313	-2.9	-4.9, -0.9	**0.012**
**fGCR**	β	95% CI	*P*-value	β	95% CI	*P*-value	β	95% CI	*P*-value	β	95% CI	*P*-value
Unadjusted	0.1	-2.9, 3.2	0.930	-1.0	-1.6, -0.4	**0.018**	-0.1	-0.9, 0.7	0.739	-0.6	-0.9, -0.2	**0.008**
Model I	0.0	-3.4, 3.5	0.980	-0.9	-1.5, -0.3	**0.044**	-0.4	-1.3, 0.5	0.415	-0.5	-0.9, -0.1	**0.013**
Model II	0.1	-3.4, 3.6	0.940	-0.9	-1.7, -0.1	0.108	-0.5	-1.5, 0.5	0.324	-0.5	-0.9, -0.1	**0.015**
Model III	0.5	-2.9, 4.0	0.760	-0.7	-1.3, 0.0	0.201	-0.5	-1.4, 0.5	0.348	-0.5	-0.9, -0.1	**0.021**

Model I adjusted for age and BMI. Model II adjusted for age, BMI, and course of disease. Model III adjusted for age, BMI, course of disease, and history of diabetes.

FCP, fasting C-peptide; fCGR, fasting C-peptide-to-glucose ratio; LCG, low-cortisol group; HCG, high-cortisol group.

P-values < 0.05 are shown in bold.

As there may be some heterogeneity in cortisol secretion and glucose metabolism between premenopausal and postmenopausal women, we conducted a stratified analysis according to menopausal status; although we did not observe a significant difference in BMI or tumor location between the two groups, the course of disease in premenopausal women with MACS was longer than that in postmenopausal patients. The level of F_00:00_ in premenopausal patients was also higher than that in postmenopausal patients, and the level of F_08:00_ and the 1-mg DST inhibition rate were lower than for postmenopausal patients (**Table 5**); after adjustment for age, BMI, and course of disease, the significant differences in these indices dissipated (**Table 6**). In terms of glucose metabolism, the level of FPG in postmenopausal patients was higher than that in premenopausal patients, but there were no significant differences in FCP, HbA1c, or fCGR (no premenopausal women were diagnosed with diabetes or used hypoglycemic drugs prior to their endocrine assessments). After adjusting for age, BMI, and course of disease, with a *P* value of 0.703, the difference in FPG between the two groups vanished. In addition, in the correlation analysis between autonomous cortisol secretion and glucose metabolism, we observed that the correlations between the 1-mg DST inhibition rate and FCP and fCGR were only apparent in premenopausal women after adjustment for age, BMI, and course of disease (**Table 7**).

**Table 5:** Clinical characteristics and biochemical indices of female patients with MACS in relation to menopausal status.

	Premenopausal	Postmenopausal	*P*-value
n	28	37	
Age (years, x ± s)	42.6 ± 7.4	56.5 ± 6.7	**<0.001**
BMI (kg/m^2^, x ± s)	25.0 ± 2.9	26.2 ± 3.7	0.181
Course of disease (months, M[Q1, Q3])	1.0 (0.5-3.5)	1.0 (0.5-2.0)	**0.043**
Tumor Location (n (%))			0.943
Left adrenal	11 (39.3%)	13 (35.1%)	
Right adrenal	12 (42.9%)	17 (45.9%)	
Bilateral adrenal	5 (17.9%)	7 (18.9%)	
ACTH_00:00_ (pmol/L, M[Q1,Q3])	1.1 (1.1-1.8)	1.1 (1.1-1.7)	0.413
ACTH_08:00_ (pmol/L, M[Q1,Q3])	1.6 (1.2-2.8)	2.5 (1.6-3.8)	0.153
ACTH_16:00_ (pmol/L, M[Q1,Q3])	1.3 (1.1-2.1)	1.4 (1.1-2.2)	0.975
F_00:00_ (nmol/L, M[Q1,Q3])	186.1 (131.4-275.6)	134.3 (97.3-232.7)	**0.047**
F_08:00_ (nmol/L, M[Q1,Q3])	365.9 (291.8-397.8)	417.4 (331.3-538.8)	**0.005**
F_16:00_ (nmol/L, M[Q1,Q3])	245.3 (170.1-302.2)	263.3 (195.7-311.3)	0.722
24h UFC (μg/24h, M[Q1, Q3])	418.8 (291.2-564.8)	404.1 (299.4-481.3)	0.965
1mg-DST ACTH (pmol/L, M[Q1,Q3])	1.4 (1.1-1.8)	1.1 (1.1-1.2)	0.531
1mg-DST F (nmol/L, M[Q1,Q3])	183.3 (111.0-298.2)	175.4 (75.7-244.9)	0.127
1mg-DST Inhibition Rate (M [Q1, Q3])	0.5 (0.0-0.6)	0.6 (0.4-0.8)	**0.008**
LDDST ACTH (pmol/L, M[Q1,Q3])	1.3 (1.1-1.6)	1.1 (1.1-1.1)	0.243
LDDST F (nmol/L, M[Q1,Q3])	202.4 (162.6-380.1)	162.5 (85.6-261.6)	0.094
FPG (mmol/L, x ± s)	4.8 ± 0.7	5.5 ± 1.8	**0.040**
FCP (mU/L, M[Q1,Q3])	2.4 (1.9-2.9)	2.6 (1.8-3.2)	0.601
fGCR (mU/mmol, M [Q1, Q3])	0.4 (0.4-0.6)	0.5 (0.4-0.6)	0.871
HbA1c (%, x ± s)	5.5 ± 0.7	6.2 ± 1.8	0.077
Diabetes (n (%))	0 (0.0%)	13 (35.1%)	**<0.001**

Continuous data are shown as mean ± standard deviation or median (interquartile range), and categorical data are shown as frequency (%). P-values < 0.05 are shown in bold.

BMI, body mass index; F_00:00_, cortisol level at 00:00; F_08:00_, cortisol level at 08:00; F_16:00_, cortisol level at 16:00; UFC, urine-free cortisol level; 1-mg DST, 1-mg overnight dexamethasone test; LDDST, low-dose dexamethasone test; FPG, fasting plasma glucose; FCP, fasting C-peptide; fCGR, fasting C-peptide-to-glucose ratio.

**Table 6:** Logistic regression used to evaluate the difference in autonomous cortisol secretion between female MACS patients of different menopausal statuses.

	F_00:00_			F_08:00_			1mg-DST F			1mg-DST Inhibition Rate		
	β	95% CI	*P*-value	β	95% CI	*P*-value	β	95% CI	*P*-value	β	95% CI	*P*-value
Unadjusted	-52.6	-103.4, -1.7	**0.047**	85.5	27.5, 143.6	**0.005**	-47.9	-108.7, 12.9	0.127	0.2	0.1, 0.4	**0.008**
Model I	-35.7	-108.6, 37.1	0.340	8.6	-69.1, 86.2	0.829	-17.7	-104.4, 69.0	0.691	0.0	-0.2, 0.2	0.741
Model II	-45.6	-119.1, 28.0	0.229	5.8	-73.8, 85.3	0.888	-24.6	-113.0, 63.9	0.588	0.0	-0.2, 0.3	0.691

Model I adjusted for age and BMI. Model II adjusted for age, BMI, and course of disease.

F_00:00_, cortisol level at 00:00; F_08:00_, cortisol level at 08:00; 1-mg DST F, cortisol post-1-mg overnight dexamethasone test.

P-values < 0.05 are shown in bold.

**Table 7:** Logistic regression used to evaluate the association between 1-mg DST inhibition rate and glucose metabolism in female MACS patients as stratified by menopausal status.

Model		Premenopausal			Postmenopausal	
FCP	β	95% CI	*P*-value	β	95% CI	*P*-value
Unadjusted	-1.7	-3.5, 0.0	0.069	-0.5	-2.7, 1.6	0.625
Model I	-2.4	-4.1, -0.8	**0.012**	-1	-2.8, 0.8	0.295
Model II	-2.5	-4.2, -0.8	**0.012**	-1.4	-3.4, 0.6	0.175
**fGCR**	β	95% CI	*P*-value	β	95% CI	*P*-value
Unadjusted	-0.3	-0.7, 0.1	0.113	-0.1	-0.5, 0.2	0.389
Model I	-0.5	-0.8, -0.1	**0.021**	-0.1	-0.5, 0.2	0.400
Model II	-0.5	-0.8, -0.1	**0.021**	-0.1	-0.5, 0.2	0.440

Model I adjusted for age and BMI. Model II adjusted for age, BMI, and course of disease.

FCP, fasting C-peptide; fCGR, fasting C-peptide-to-glucose ratio.

P-values < 0.05 are shown in bold.

## Discussion

Similar to the situation for primary aldosteronism, scholars have not reached a consensus on the diagnostic criteria for SCS or MACS. In 2009, the American Association of Clinical Endocrinologists (AACE) and American Association of Endocrine Surgeons (AAES) medical guidelines recommended three screening tests for SCS evaluation: a midnight salivary cortisol level, 24 h UFC, and 1-mg DST (16). In addition, the guidelines clearly indicated that a 1-mg DST was better than a 24 h UFC in AI screening. Therefore, in order to improve the specificity of diagnosis, AACE/AAES recommended that the cut-off point for serum cortisol after a 1-mg DST be 138 nmol/L. In 2016, a guideline jointly issued by the ESE and the European Network for the Study of Adrenal Tumors (ENSAT) recommended a 1-mg DST as the first-line screening test, emphasizing that the concept of “yes/no” be abandoned when interpreting the results of a 1-mg DST, and that the serum cortisol level after a 1-mg DST be regarded as a continuous spectrum from normal secretion to mild secretion and then to excessive secretion (15). However, despite the fact that some researchers believe the 50 nmol/L cutoff value is insufficiently sensitive (17), for the convenience of clinical practice, the guideline still recommended that MACS be excluded if the 1-mg-DST F was lower than 50 nmol/L, that MACS be considered if the 1-mg-DST F was between 50 nmol/L and 138 nmol/L, and that further tests be conducted to clarify the diagnosis when the 1-mg-DST F was higher than 138 nmol/L. The guidelines’ authors also determined that further examination and evaluation for metabolic abnormalities are recommended for individuals with a 1-mg-DST F higher than 50 nmol/L. Based on the aforementioned recommendations, the cortisol-related inclusion criteria of all of our MACS patients were ultimately determined to constitute a disordered circadian rhythm of cortisol secretion, with a 1-mg-DST F >50 nmol/L and LDDST F levels >50 nmol/L. Thus, 98 patients were finally included in the study, with a male-to-female ratio of 1:1.9, which was similar to the results of our previous research (a male-to-female ratio of 1:2.38) (18) and the work from Comlekci (with a male-to-female ratio of 1:2.41) (14).

In our study, we ascertained that those indices that exhibited a better indicative performance for autonomous cortisol secretory capability (such as F_00:00_, 1-mg-DST F, and LDDST F) were significantly higher in females than in male MACS patients, and that the inhibition rate of 1-mg DST was lower in women than in men. This difference was still significant after adjusting for age, BMI, and course of disease, which clearly suggests that female patients with MACS produced more robust autonomous cortisol secretion. However, the specific reason for this remains unknown. We speculate that this may be related to the following scenario. Steroid receptors are a subfamily of the nuclear receptor superfamily, and steroid hormones play a role in physiological states by binding to intracellular steroid receptors. The steroid receptor family includes glucocorticoid receptor, progesterone receptor, estrogen receptor, androgen receptor, and mineralocorticoid receptor, which all possess high sequence identity and a similar mechanism of action (19). The ligand-binding domain of the cortisol receptor demonstrates 55% sequence identity with the progesterone receptor and 30% with the estrogen receptor (20); this therefore provides progesterone and estrogen with a certain degree of glucocorticoid-like activity, and an even higher affinity than cortisol for some glucocorticoid-receptor subtypes (21). Therefore, estrogen and progesterone may combine and occupy cortisol receptors, resulting in an increase in free cortisol levels. Estrogen can augment the synthesis of corticosteroid-binding globulin (CBG) in the liver, leading to an elevation in total serum cortisol. And since the kinetics of cortisol binding to CBG is not linear, sex differences in CBG could also be responsible for differences in F00:00 between men and women, even though other time points are not different. Unfortunately, due to constrained conditions, we are unable to identify the level of CBG or salivary cortisol. In addition, investigations have revealed that estrogen and progesterone receptors are related to the growth of tumor cells (22).

Although SCS is not associated with the classical features of Cushing’s disease by definition, there is clear evidence to suggest long-term consequences of mild cortisol excess, such as glucose-metabolism disorders, obesity, osteoporosis, and an impaired quality of life (10). A study by Miomira et al. indicated that there was a significant difference in insulin sensitivity between subjects with MACS and healthy controls, and that MACS patients possessed a significantly higher prevalence of impaired glucose tolerance and a greater area under the curve for glucose than did subjects with nonfunctional adrenal incidentalomas (7). As a result, some researchers pointed out that diabetic patients should be selected for MACS screening (23). However, little is known as to the differences in the effects of increased cortisol on glucose metabolism between male and female MACS patients. In the present study, although we noted no significant differences in glucose metabolism-related indicators such as HbA1c and FPG between men and women with MACS, the associations between FCP/fCGR and the cortisol-inhibition rate showed significant sex differences. This association, however, was only reflected in the female of the high cortisol group and in premenopausal individuals; that is, in this population and with the reduction in the autonomous cortisol secretion level, FCP and fCGR decreased commensurately. Evidence has also shown that FCP (24, 25) and fCGR (26–28) can indicate the degree of insulin resistance. Patel’s retrospective cohort study (25) of 5153 participants aged 40 to 74 with a fasting glucose ≥70 mg/dL and without diabetes by history or laboratory testing showed that serum C-peptide levels could serve as a marker of insulin resistance and to predict cardiovascular and overall death better than other known insulin resistance measures such as fasting plasma glucose, fasting serum insulin, and HOMA-IR. When compared with the lowest C-peptide quartile, subjects in the highest quartile manifested significantly higher adjusted hazard ratios of cardiovascular death and overall mortality after controlling for confounders. FCGR is a measure of the amount of insulin secreted for a corresponding glucose level, and is considered a simplified index in the evaluation of insulin resistance compared with the euglycemic hyperinsulinemic clamp, which was regarded as the previous “gold standard”. In the study by Wang (28) and some other investigators (26, 27, 29), the fCGR index significantly increased in individuals with insulin resistance, and it was reported to present satisfactory specificity and sensitivity compared with euglycemic hyperinsulinemic clamps and HOMA indices. The results of our study therefore suggest that the level of insulin resistance in female MACS patients improves with the decline in autonomous cortisol secretion, and that this relationship only appears in premenopausal women with MACS and who manifest overt increases in autonomous cortisol secretion. As to the reason for functional sexual dimorphism in the correlation between cortisol and glucose metabolism, we considered potential imbalances in many unknown interfering factors such as smoking, drinking, snoring, or irregular sleep habits. Female MACS patients who exhibited a lower frequency of the above factors also presented with a simpler relationship between cortisol and glucose metabolism. This phenomenon appears to explain to a degree the higher autonomous cortisol secretion that we observed for female MACS patients relative to men, but a lack of any worsening glucose-metabolic dysfunction than in male MACS patients. With regard to the differences in cortisol and glucose metabolism between premenopausal and postmenopausal women, we posit that it may relate to age impacts on glucose metabolism in postmenopausal women that exceed the effects of excessive cortisol secretion.

We appreciate that there are sex differences in the incidence rates, clinical manifestations, and complications of some endocrine diseases, and that these differences can outline the characteristics of the disease, help doctors to better understand the disease, and guide diagnosis and treatment. Through the analysis of MACS patients at our center, we described the possible sex differences in cortisol secretion level and other clinical manifestations of the disease. Although the sample size of our study was limited, and a control group of individuals with non-functioning adrenal tumors was not included (since this is a retrospective study concentrating on sex differences), we revealed one aspect of the disease *via* an analytical perspective to which few scholars currently pay attention. Except for the limited sample size and potential resulting bias, there were some other limitations that merit emphasis. Even though in the current study we adjusted a number of potential confounders, we could not rule out the possibility that our results were affected by other variables that were not included in the analyses. As some subjects were excluded in terms of basic baseline characteristics, this may also have caused a certain degree of bias. Multi-center, large-scale, and long-term prospective studies are therefore still required in the future to provide further clinical evidence.

## Conclusions

We herein made the novel observation that the autonomous cortisol secretion level of female MACS patients was higher than that for male MACS patients, and that the association between autonomous cortisol secretion levels and abnormal glucose metabolism was stronger in premenopausal MACS patients with high cortisol secretion. Therefore, this population may require closer long-term follow-up and may be suitable for more active treatment.

## Data Availability Statement

The original contributions presented in the study are included in the article material. Further inquiries can be directed to the corresponding authors.

## Ethics Statement

The study was approved by the ethics committee of Chinese PLA General Hospital (NO. S2021-555-01). A waiver of the requirement to obtain informed consent from the study subjects was approved considering the minimal risk of the study.

## Author Contributions

WG, YM and YY conceived and designed this research. RO, YY, WS, JW, LZ, KC, JD, ZL and JTD performed the data acquisition, analysis, and interpretation. RO and YY wrote the manuscript. ZL, JTD, YM and WG reviewed the full text. All the authors agreed with the final version of the manuscript.

## Conflict of Interest

The authors declare that the research was conducted in the absence of any commercial or financial relationships that could be construed as a potential conflict of interest.

## Publisher’s Note

All claims expressed in this article are solely those of the authors and do not necessarily represent those of their affiliated organizations, or those of the publisher, the editors and the reviewers. Any product that may be evaluated in this article, or claim that may be made by its manufacturer, is not guaranteed or endorsed by the publisher.
